# Research progress of platelets in neurodegenerative diseases

**DOI:** 10.3389/fnagi.2025.1544605

**Published:** 2025-06-10

**Authors:** Yu Lan, Jun Ding, Tian Yu, Chi Cheng

**Affiliations:** ^1^Department of Anesthesiology, Affiliated Hospital of Zunyi Medical University, Zunyi, China; ^2^Key Laboratory of Anesthesia and Organ Protection of Ministry of Education (In Cultivation), Zunyi Medical University, Zunyi, China; ^3^Guizhou Key Laboratory of Anesthesia and Organ Protection, Zunyi Medical University, Zunyi, China

**Keywords:** platelets, neurodegenerative diseases, neuroinflammation, antiplatelet drugs, protein aggregation

## Abstract

Neurodegenerative disease (NDD) is a disease state characterized by the loss of neuronal cells in the brain and spinal cord, including Alzheimer's disease (AD), Parkinson's disease (PD), and multiple sclerosis (MS). They have become a major challenge for the world's health system in the twenty-first century, with an increasing incidence year by year, complex and diverse causes, and a lack of effective therapeutic. The brain and spinal cord are composed of neurons, and activated platelets are highly similar to neurons. The occurrence and development of these diseases are often accompanied by platelet activation, suggesting that platelets play an important role in the pathological process of NDDs. This article reviews the research progress of platelets in common NDDs, and elaborates on the mechanisms of platelets' involvement in NDDs and the use as a therapeutic option for NDDs to providing new ideas for the diagnosis and treatment of NDDs.

## 1 Introduction

As the population ages, NDDs become more common. Globally, nearly 44 million people have Alzheimer's disease (AD) or a related form of dementia, and 8.5 million have Parkinson's disease (PD). According to data released by the World Health Organization (WHO) in September 2022, more than 55 million people worldwide are living with dementia, and this number is projected to nearly double every 20 years, increasing to 78 million by 2030 and 139 million by 2050 (Alkahtani et al., 2023). In order to alleviate the physical and psychological burden of NDDs patients and reduce the economic burden of the whole society, it is necessary to explore methods for preventing, alleviating, treating, and diagnosing NDDs. NDDs is caused by the loss of neurons or myelin sheaths, which may lead to mild neurological symptoms such as impaired motor, sensory, visual, language, and cognitive functions, and eventually develop into coma or even brain death. Common NDDs include Alzheimer's disease (AD), Parkinson's disease (PD), multiple sclerosis (MS), and other conditions. The pathogenesis of these diseases is complex. It is currently believed that pathological protein aggregation, disruption of the neurovascular unit, and neuroinflammation ultimately lead to neuronal death, which are common pathological features of NDDs (Wilson et al., 2023; Liu et al., 2023). More and more studies suggest that platelet activation plays an important role in these characteristic pathological changes and platelets can also express, store, and secrete neuron-specific proteins, such as amyloid precursor protein (APP), amyloid β-protein (Aβ), tau, α-synuclein, brain-derived neurotrophic factor (BDNF), monoamine oxidase (MAO), reelin, and ephrin (Beura et al., 2022a,c), which can be used as a good peripheral model for NDDs and are widely studied in various NDDs. Therefore, this review summarizes the research progress of platelets in common NDDs, expounds the pathological mechanisms of activated platelets participating in NDDs by promoting pathological protein aggregation, disruption of neurovascular units (blood-brain barrier disruption, microvascular dysfunction), and neuroinflammation, and discusses the advantages and feasibility of platelets in the diagnosis and treatment of NDDs.

## 2 Structure and function of platelets

Platelets, the smallest blood cells in the human body, have an average diameter of 2–4 μm, with a normal range of (150–350) × 10^9^/L in healthy adults, and a lifespan of ~7–10 days. Most aging platelets are cleared in the spleen. The anatomical structure of platelets can generally be divided into four main regions from outside to inside: the peripheral zone, the sol-gel zone, the organelle zone, and the membrane zone. The peripheral zone is primarily composed of the extracellular coat (glycocalyx) and the cell membrane, rich in glycoproteins (GPs) necessary for platelet adhesion, activation, and aggregation. The most abundant among these is the GP IIb/IIIa complex, a Ca^2^-dependent dimeric complex. The sol-gel zone serves as the matrix of the platelet cytoplasm and is rich in various microtubules and microfilaments, playing an essential role in maintaining platelet shape, release reactions, and contraction activities. The organelle zone contains platelet granules (such as α granules, dense granules, and lysosomal granules), electron-dense objects, peroxisomes, lysosomes, and mitochondria. The membrane zone primarily includes the open canalicular system and dense tubular system, where the open canalicular system serves as a pathway for exchange between platelets and plasma components, and the dense tubular system acts as a storage site for Ca^2^, responsible for the synthesis of thromboxane A_2_, regulating the contraction activity of platelet contractile proteins and platelet release reaction (Gremmel and Frelinger, 2024).

Traditional views hold that platelets are only involved in hemostasis and thrombosis; however, only 1/10th of circulating platelets is required for hemostasis, indicating that platelets have other functions. Under normal circumstances, platelets are in a resting state, mainly responsible for maintaining blood fluidity. However, once environmental changes occur, platelets respond as highly reactive and secretory cells by undergoing structural changes and releasing various chemokines and cytokines. Numerous studies indicate that platelets express various receptors on their surface that can recognize pathogen-associated molecular patterns (PAMPs) and damage-associated molecular patterns (DAMPs). Besides, platelets can directly interact with immune cells, such as neutrophils, lymphocytes, monocytes, and macrophages, facilitating the activation, polarization, migration, and cytokine secretion of these cells. Therefore, platelets also play an essential role in regulating inflammation and immunity (Koupenova et al., 2018).

## 3 Platelets and neurons

Despite differences in embryonic origin, function, and localization between platelets and neurons, there are many similarities between them. Firstly, the α granules of platelets are comparable to the large and dense core synaptic vesicles of neurons, while dense granules are akin to the small and dense core synaptic vesicles found in neurons (Leiter and Walker, 2020). Secondly, platelets store and secrete abundant neurotransmitters, including serotonin, dopamine, glutamate, γ-aminobutyric acid, and other biogenic amines, which are crucial for transmitting peripheral environmental information to the brain and play a vital role in intercellular communication between neurons. Thirdly, the mode of information transfer between platelets and neurons is similar. Neurons transfer neurotransmitters released from the presynaptic membrane to the postsynaptic membrane through the influx of calcium ions, facilitating communication between neurons; whereas platelets also involve calcium-dependent activation when they receive external stimuli (such as damage signals), releasing signaling molecules via vesicles that bind to specific receptors on target cells to execute their functions and this receptor-ligand interaction is central to information transfer (Ponomarev, 2018; Burnouf and Walker, 2022). In addition to transmitting information, platelets can directly interact with Neural lipid rafts (NLR), resulting in the release of 5-OH, which stimulates the formation of dendritic spines and induces the expression of neuronal early response genes and synaptic plasticity genes; platelets can also directly release cytokines (e.g., BDNF; Bouhaddou et al., 2024) to promote neuronal growth and synaptic formation, thus enhancing synaptic plasticity; In addition to direct effects, platelets can also regulate neuroinflammation and indirectly affect neuronal electrical activity and synaptic plasticity by releasing substances such as platelet activating factor (PAF) and 5-OH (Dukhinova et al., 2018; Kopeikina and Ponomarev, 2021).

## 4 Platelets and neurodegenerative diseases (NDDs)

The high degree of similarity between platelets and neurons provides a favorable basis for studying the link between platelets and NDDs. Several proteins are expressed in neurons and platelets, even at abnormally high concentrations in platelets (Canobbio, 2019). BDNF is widely distributed throughout the nervous system, and platelets are the most important reservoir of BDNF. In NDDs such as AD, PD, and MS, BDNF expression is decreased, which may be associated with interfering with synaptic plasticity, disrupting dopaminergic signaling pathways, inhibiting neuronal activity, and promoting inflammatory immune responses (Bouhaddou et al., 2024). Ephrin, a related ligand for Erythropoietin-producing hepatomocellular (Eph) receptors. A large number of Eoh receptors and their ephrin ligands are distributed in synapses, and upon ligand stimulation, Eph receptors near the membrane undergo tyrosine phosphorylation and participate in synapse formation. Synaptic loss, accompanied by alterations in synaptic protein composition and function, usually occurs as early as the first stages of neurodegenerative disease and is thought to be the best pathologic correlate of cognitive decline (Shu et al., 2023).

### 4.1 Platelets and Alzheimer's disease (AD)

AD is the most common neurodegenerative disease, and its incidence increases exponentially with age. The main clinical symptoms of AD include gradual loss of episodic memory, as well as cognitive and behavioral impairments. The typical pathological features mainly include senile plaques (SPs) formed by extracellular Aβ deposition and neurofibrillary tangles (NFTS) formed by intracellular tau protein hyperphosphorylation. This aggregation of Aβ and Tau adversely affects synaptic plasticity and triggers neuronal cell death (Scheltens et al., 2021). Platelets contain 90% of the circulating APP and all the enzymes involved in APP metabolism (Li and Liu, 2022; Catricala et al., 2012), and once activated, they can secrete a large amount of Aβ into the bloodstream, which can be actively transported to the brain through the blood-brain barrier (BBB), promoting Aβ deposition (Carbone et al., 2021). Platelets release Aβ1–40 and Aβ1–42 in response to hemostatic, immune, and hypoxic stimuli, and show the release of the more fibrillogenic and pathogenic Aβ1–42 (Wolska et al., 2023). Wu injected platelets and plasma from AD mice of different ages into 10-week-old C57 mice. The platelets and plasma from 3- and 6-month-old AD mice did not lead to Aβ deposition in the brains of recipient mice, but the platelets from 10- and 15-month-old AD mice caused significant Aβ deposition in the brains of 75% and 100% of recipient mice, respectively. However, only 25% of mice with plasma-induced significant Aβ deposition in the brain. This indicates that as the age of the donor mouse increases, the deposition of Aβ in the recipient mouse's brain increases, and the effect of platelet injection is more significant than plasma injection (Wu et al., 2021). Tau mainly regulates axonal transport by affecting the polymerization and stabilization of microtubules, and is therefore closely related to establishing and maintaining neuronal morphology and function (Gendron and Petrucelli, 2009). The presence of tau in platelets was first suggested in a study by Neumann et al. and the analysis of different tau fractions in platelets could serve as a new biological marker for AD (Neumann et al., 2011). The ratio of high molecular weight tau/low molecular weight tau in platelets correlates with localized brain atrophy in patients with AD (Slachevsky et al., 2017). Interestingly, Aβ accumulation promotes the aggregation of Tau, which in turn plays an integral role in Aβ-induced synaptic defects (Chen et al., 2012). Glycogen synthase kinase-3 (GSK-3) is a highly conserved protein serine/threonine kinase found in platelets in mammals with α and β isoforms. GSK-3β is expressed in almost all brain regions and is an important hub that controls neuronal signaling pathways (Chen et al., 2012). It can participate in the development of AD. GSK-3β enhances β-secretase 1 (BACE1) activity and mediates the toxicity of Aβ aggregates (Ly et al., 2013). GSK-3β is one of the major kinases responsible for tau phosphorylation, and elevated pathogenic tau phosphorylation is also evident in regions where GSK-3β activity is upregulated (Albeely et al., 2022). More interestingly, Aβ can activates GSK-3, leading to increased hyperphosphorylation of tau (Nguyen et al., 2020), and GSK-3 serves as a molecular link between Aβ and Tau (Sayas and Ávila, 2021). Platelets contain two isoforms of GSK-3: GSK-3α and GSK-3β and activated platelets affect the activity of GSK-3β by promoting its phosphorylation at the Ser9 site, thereby influencing the course of AD (Barry et al., 2003). Reelin is a signaling protein that regulates synaptic function and plasticity in the mature brain and plays an important role in the pathology of AD. Reelin protects the brain from Aβ-induced synaptic dysfunction and memory impairment (Lane-Donovan et al., 2015) while linking Aβ and tau phosphorylation dysregulation that Aβ induces impaired Reelin signaling, resulting in a reduced ability of Reelin to down-regulate tau phosphorylation via GSK3β (Cuchillo-Ibáñez et al., 2013). As early as 1980, Adolfsson et al. (1980) found that the activity of MAO-B in the brain and platelets of AD patients increased. A study found a substantial association between Mini-mental State Examination (MMSE) score and MAO-B activity, and a positive correlation between MMSE score and platelet MAO-B activity in AD patients, suggesting that more severe AD symptoms were associated with lower MAO-B activity (Muck-Seler et al., 2009). In summary, platelets express and store APP, Aβ, tau, GSK-3, reelin and MAO-B, making them a suitable peripheral model for studying the pathophysiology of AD (Veitinger et al., 2014).

With the pursuit of non-invasive diagnostic AD, peripheral diagnostic markers have attracted attention. Oberacher et al. analyzed 26 healthy controls (CO), 20 patients with mild cognitive impairment (MCI), and 26 patients with AD for platelet phosphatidylcholines (PC) metabolomics and found that lipid PC ae C40:4 significantly differentiated between AD from CO, whereas other lipids (PC aaC32:0, PC ae C32:2, and PC aeC34:1) distinguished MCI from CO; and a 9-month follow-up study of 10 patients found that PC ae C40:4 < 0.30 μM could be used to assist in the diagnosis of AD (Oberacher et al., 2017). Using proteomic techniques, Yu et al. determined that the combination of mitochondrial dysfunction-associated protein:Prohibitin (PHB), ubiquinol-cytochrome c reductase hinge protein (UQCRH) and platelet activation-associated protein:Glycoprotein Ib Platelet Subunit Alpha (GP1BA), FINC could most accurately identify cognitive decline in patients with MCI and AD and these proteins are positively correlated with MMSE scores, suggesting that platelet markers have early diagnostic value in AD (Yu et al., 2021). Another study found a significant negative correlation between platelet large cell ratio (P-LCR) and Montreal Cognitive Assessment (MoCA) scores in patients with AD carrying the APOE ε4 allele, and the rate of change in P-LCR tended to increase with disease progression, which in general means that P-LCR is associated with an increased risk of AD and increases with disease progression in AD patients (Fu et al., 2024). Researchers longitudinally followed-up 154 cases of dementia, including 121 cases of AD, and found that individuals who did not receive antiplatelet therapy and had high platelet reactivity had a higher risk of developing dementia in later life, highlighting the role of platelet function in the risk of AD. This suggests that platelet phenotype may be associated with the incidence of dementia and may have prognostic value (Ramos-Cejudo et al., 2022). Through the above studies, we can see that platelets not only play a key role in the pathogenesis of AD, but may also be an important target for diagnosis and treatment. Future studies should further explore the association between platelet-related biomarkers and Alzheimer's disease in order to better understand the mechanism and optimize clinical applications.

### 4.2 Platelets and Parkinson's disease (PD)

PD is the second most common degenerative disease, which causes motor and non-motor dysfunction due to the degeneration and loss of dopaminergic neurons in the substantia nigra pars compacta and striatum, as well as the formation of neuronal Lewy bodies. The misfolding, structural changes, and abnormal aggregation of α-synuclein lead to the formation of Lewy bodies and the damage of dopaminergic neurons, which is key protein responsible for Parkinson's disease (Bloem et al., 2021). In addition to the Central Nervous System (CNS), α-synuclein is also widely expressed in the blood, with platelets being its main host (Acquasaliente et al., 2022). Apart from α-synuclein, platelets also express tyrosine hydroxylase (TH), vesicular monoamine transporter 2 (VMAT2) and dopamine transporter (DAT; Beura et al., 2022a,c). Circulating dopamine is concentrated in the cytoplasm of platelets through DAT, and then further absorbed into the dense granules for storage until secretion upon platelet activation (Osinga et al., 2016). TH is a key enzyme in the production of dopamine, and although there are no definitive studies demonstrating the changes of TH, a decrease in VMAT2 mRNA levels in PD platelets (Sala et al., 2010) and a decrease in the amount of dopamine stored in vesicles leads to a decrease in intracellular dopamine concentration, which may inhibit TH activity. In patients with PD, platelet activation (Adams et al., 2019), and mitochondria are the main damaged organelles, with reduced respiratory rate and ATP production, affecting the normal function of platelets (Ferrer-Raventós and Beyer, 2021). Parkin and PTEN-induced kinase-1 (PINK1) are PD-specific proteins that mainly affect mitochondrial function. Compared to other tissues, Parkin is highly expressed in platelets and contributes to autophagy, especially mitochondrial autophagy (Lee et al., 2020). PINK1 is a mitochondrial enzyme that can potentially regulate platelet activation, and its deficiency may significantly increase platelet activation and thrombosis. PINK1-triggered mitochondrial autophagy can remarkably reduce platelet oxidative stress (Walsh et al., 2018). Therefore, platelets can be used as a valuable peripheral model for PD (Beura et al., 2022a,c).

The relevant indexes of platelets can predict the occurrence of PD: Through Linkage Disequilibrium Score Regression, based on common genetic variations between platelet parameters and PD risk, the genome-wide coheritability was tested, and it was found that there was a significant genetic correlation between platelet distribution width (PDW), an index of platelet size variability, and PD risk (Tirozzi et al., 2020).

### 4.3 Platelets and multiple sclerosis (MS)

MS is considered to be the most common autoimmune disease, a chronic inflammatory demyelinating disease of the CNS (Kalinowska-Łyszczarz et al., 2022). Its main characteristics are multifocal and asymmetric white matter lesions in the CNS, leading to neurological dysfunction and causing different clinical symptoms, such as visual complications, gait imbalance, muscle spasms, persistent numbness, and cognitive deficits (Javalkar et al., 2016). As a multifactorial disease, the underlying cause of MS is unknown. Platelet activation has been observed in patients with MS, and activated platelets in the brain may provide an additional source of reactive oxygen and nitrogen species (ROS/RNS), which lead to oxidative stress, further exacerbating neuronal and glial cell damage with demyelination (Wachowicz et al., 2016). Experimental autoimmune encephalomyelitis (EAE) is a well-recognized animal model of multiple sclerosis (MS) in mice. Orian's research group found that the increase in circulating platelets occurred earlier than the appearance of clinical symptoms in EAE and the extravasation of inflammatory cells (Sonia D'Souza et al., 2018). However, the increase of platelets is not limited to the periphery, and platelets have also been found to invade the CNS of MS patients and EAE mice. This phenomenon has not been observed in healthy individuals and mice (Langer et al., 2012), indicating that platelets play an important role in the pathogenesis of MS/EAE.

Platelet RNA can be used as a new blood biomarker for MS. Researchers isolated platelets and performed RNA-seq detection, and found that compared with healthy people, MS caused 1,249 changes in platelet RNA profiles, including increased expression of Epithelial-Stromal Interaction 1 (EPSTI1) and IFN alpha inducible protein 6 (IFI6), and decreased expression of Ribosomal protein S6 kinase A3 (RPS6KA3), which is consistent with the reported inflammatory characteristics in the blood of MS patients. The RNAs were subsequently used as input for a MS classifier, capable of detecting MS with 80% accuracy in the independent validation series (Sol et al., 2020). The RPS6KA3 has been shown to be downregulated in the blood from MS patients in remission (Achiron et al., 2018). In addition, the platelet signaling pathway CD40/CD40L plays a crucial role in many various autoimmune diseases, including MS, and is considered a marker of the early stages of autoimmune inflammation. More than 95% of soluble CD40 ligand (sCD40L) in plasma comes from the granules of activated platelets (Aloui et al., 2016).

## 5 Mechanism of platelets participating in NDDs

In addition to promoting pathologic protein aggregation, platelets can also contribute to NDDs through pathological changes in the neurovascular unit (NVU) and neuroinflammation.

### 5.1 Pathological changes in neurovascular unit

NVU refers to a structurally interconnected whole that includes neurons, glial cells (such as oligodendrocytes, microglia, and astrocytes), and vascular constituents (including endothelial cells, pericytes, smooth muscle cells, and the basement membrane). It is the most basic functional unit present in both the brain and peripheral regions (Yu et al., 2020). NDDs are associated with factors such as dysfunction of cerebral microvasculature, disintegration of the neurovascular unit, and impairment of the BBB.

#### 5.1.1 Impact on the blood-brain barrier (BBB)

The BBB is a highly selective biological barrier primarily composed of microvascular endothelial cells, astrocytes, and pericytes, which prevents various harmful substances entering the brain from the blood. In most NDDs (such as AD, PD, and MS), platelets are activated. Activated platelets significantly affect the integrity of the BBB by releasing platelet-activating factor, P-selectin, ADP, platelet-derived growth factor, Aβ, inflammatory factors, and forming platelet-neutrophil aggregates, thus releasing ROS that damage endothelial cell DNA, proteins, and lipids (Lv et al., 2023). In MS patients, activated platelets can produce IL-1α, triggering endothelial activation and the release of sCD40L and PF4/CXCL4, leading to altered BBB permeability (Wachowicz et al., 2016). Increased platelets in aging AD mouse models promote apoptosis of epithelial cells, resulting in increased permeability of *in vitro* BBB models (Wu et al., 2021). Kopeikina et al. showed that platelets actively secrete 5-OH by interacting with NLR in the CNS, leading to increased BBB permeability. Interestingly, platelet-derived but not CNS-derived 5-OH enhances neuronal electrical activity, and platelets also enhance neuronal electrical activity by upregulating the expression of genes associated with inflammation (IL-1β and TNF), early neuronal response genes (*EGR1* and *ARC*), and oxidatively phosphorylated mitochondrial genes (*MT-CO1, MT-ATP6*, and *MT-ND6*) activity as well as pro-inflammatory and oxidative stress, further leading to increased BBB permeability (Kopeikina et al., 2020).

#### 5.1.2 Pathological changes in microvasculature

In many cases, typical neurodegenerative markers coexist with vascular pathologies, such as microhemorrhages, microinfarcts, and small artery sclerosis (Iadecola et al., 2019). Platelets are cells that initiate and accelerate the process of vascular inflammation, which is crucial for the health of cerebral blood vessels. In NDDs, platelets infiltrate the brain parenchyma, accompanied by the release of matrix metalloproteinases derived from platelets, thereby promoting vascular damage (Kniewallner et al., 2018). When vessels are damaged, activated platelets release a series of coagulation and vasculature-related factors; this release not only impacts the integrity of blood vessels but may also lead to dysfunction of microvasculature, further exacerbating the pathological progression of NDDs. For example, vascular pathology in AD patients includes lesions in cerebral microcirculation, dysfunction of the neurovascular unit, a pro-coagulation state, and hypertensive vascular remodeling (Cortes-Canteli and Iadecola, 2020). Furthermore, decreased cerebral blood flow and glucose metabolism, along with increased cerebrovascular resistance, have been observed in both human AD cases and transgenic mouse models overexpressing amyloid precursor protein, as well as in high-risk populations for AD carrying the apolipoprotein E4 allele (Korte et al., 2020). This may be due to activated platelets releasing Aβ and interacting with Aβ (García-Culebras et al., 2024), leading to platelet adhesion and aggregation to damaged brain microvessels, releasing ADP, thromboxane A2 (TXA2), serotonin, and other substances that stimulate leukocyte recruitment to affected sites, resulting in exacerbated inflammation and thrombosis, ultimately damaging the structural and functional integrity of blood vessels.

On the other hand, cardiovascular risk factors can lead to long-term pathological vascular damage, such as endothelial dysfunction, atherosclerosis, changes in vascular reactivity, and insufficient cerebral blood perfusion, among various common cellular and molecular pathological changes that may be associated with NDDs (Iadecola et al., 2019). Cardiovascular diseases and risk factors are associated with continuously persistent chronic inflammatory states (Furman et al., 2019), which activate the immune system (Rohde et al., 2022). Recent research evidence suggests that immune system activation plays a crucial role in neurodegeneration (Endres et al., 2022), with platelets serving as an important mediator of immune regulation.

### 5.2 Promotion of neuroinflammation

Neuroinflammation refers to a highly complex response of the CNS to certain stimuli such as trauma, infection, and NDDs. This involves immune responses of cells, activation of glial cells, release of inflammatory mediators, and synthesis of ROS/RNS. Within the CNS, there exist new ligands that are recognized by platelets, which are located in lipid rafts of astrocytes and neurons. These new platelet-recognized ligands play a crucial role in the induction and maintenance of inflammation in the CNS (Sotnikov et al., 2013). Platelets are small enough to traverse the microcapillaries of the brain under both healthy and pathological conditions, entering the CNS (Burnouf and Walker, 2022). They interact with the gangliosides of neuronal axons and astrocytes, prompting the release of serotonin from platelet dense granules, stimulating neuronal electrical activity, enhancing oxidative stress, and inducing the production of pro-inflammatory factors IL-1β and TNF, thereby accelerating the progression of NDDs (Kopeikina et al., 2020; Kniewallner et al., 2020). Besides, through the CD40L on the surface of platelets and NFκB signaling within glial cells, the pro-inflammatory actions of microglia and astrocytes can be promoted, leading to neuroinflammation (Kong et al., 2023). Studies have shown that platelets bind to sialated glycosphingolipids (gangliosides) integrated into brain lipid rafts via multiple receptors with P-selectin (CD62P), leading to platelet activation and degranulation. Interactions between platelets and brain lipid rafts result in activation and secretion of pro-inflammatory cytokines (IL-1), chemokines (CXCL4/PF4), and serotonin, which attract immune cells to the lesion site and activate them *in situ* (Sotnikov et al., 2013). The initial interaction of P-selectin on platelets with P-selectin glycoprotein ligand-1 (PSGL-1) on neutrophils plays a central role in neutrophil recruitment, and the formation of neutrophil extracellular traps (NETs). Platelets also indirectly regulates leukocyte recruitment and activation through fibrinogen or von Willebrand factor (VWF). Inflammatory factors, chemokines and growth factors, such as PF4, IL-1, Platelet-derived growth factor (PDGF), PAF, TXA2, 5-OH, amplify platelet-dependent neutrophil recruitment, which further promotes neutrophil recruitment, adhesion, and survival. On the other hand, the interaction of activated neutrophils with platelets leads to increased ROS production and NETs formation, and in turn, ROS and NETs support platelet-neutrophil interactions, platelet activation, and vascular inflammation, further amplifying the inflammatory response (Rayes et al., 2020). The production of ROS is also a major contributor to neurodegeneration triggered by neuroinflammation during NDDs (Beura et al., 2022a,b). However, the mechanisms by which platelets produce ROS remain elusive. Healthy platelets possess an intact ubiquitin-proteasome system (UPS), enabling various platelet functions, but under pathological conditions, damage to the UPS is linked to increased ROS production (El-Kadiry and Merhi, 2021).

**Figure 1:**
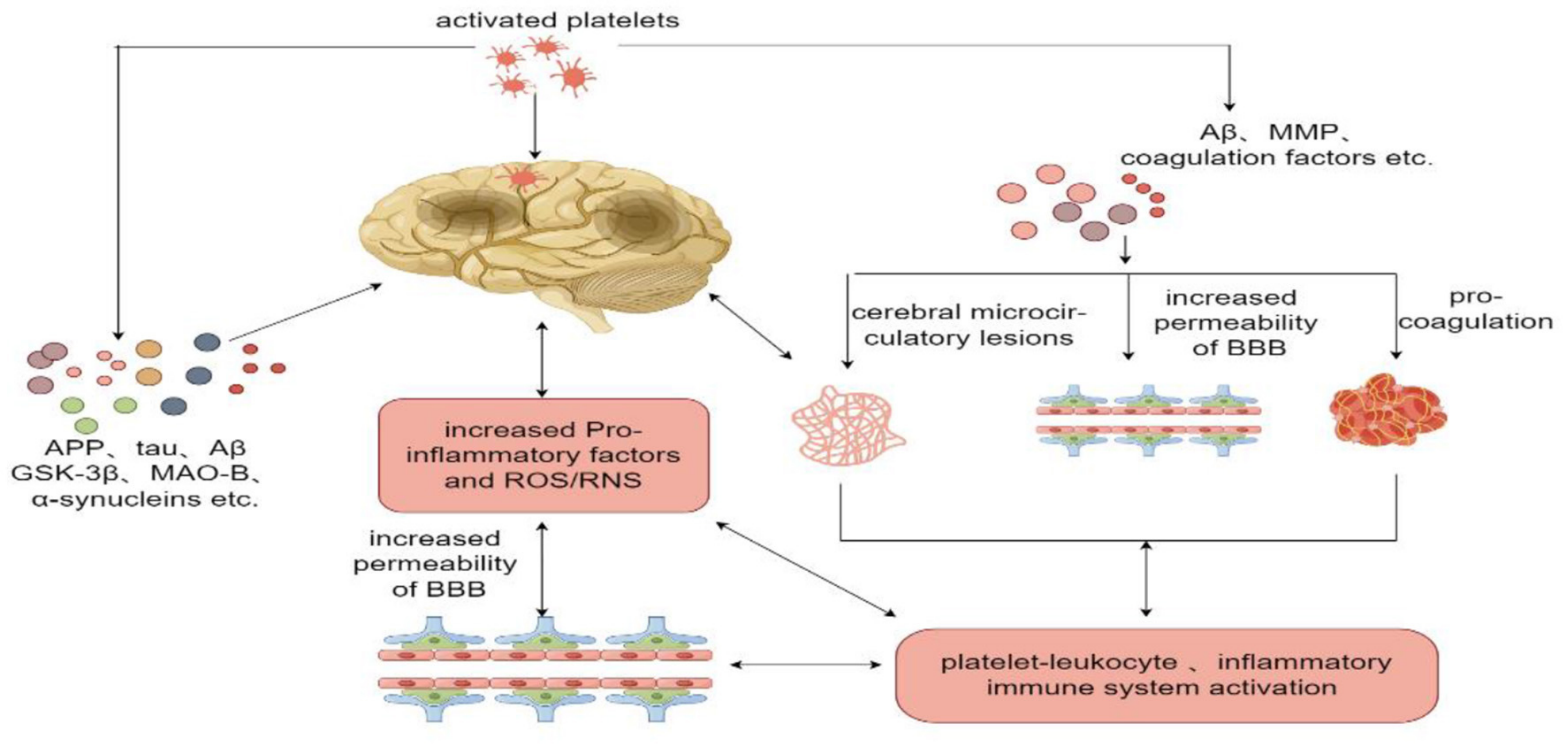
On NDDs, platelets activate and enter the CNS, where they interact with neuroglia and NLR, releasing pro-inflammatory factors and enhance oxidative stress; in addition, activated platelets also release a large number of proteins and factors, leading to pathological protein aggregation, increased permeability of BBB, cerebral microvascular lesions, platelets-leukocytes interplay and inflammatory immune cell activation, which complement each other and jointly promote the pathological process of NDDs. Figure was created with Figdraw. Mechanism of platelets participating in NDDs.

To sum up, in NDDs, platelets activate and enter the CNS where they interact with neuroglia and NLR, releasing pro-inflammatory factors and enhance oxidative stress; in addition, activated platelets also release a large number of proteins and factors, leading to pathological protein aggregation, increased permeability of BBB, cerebral microvascular lesions, platelets-leukocytes interplay and inflammatory immune cell activation, which complement each other and jointly promote the pathological process of NDDs (Figure 1).

## 6 Platelets as a therapeutic approach for NDDs

### 6.1 Antiplatelet drugs and NDDs

The pathological characteristics of NDDs primarily revolve around neuronal damage in the brain and spinal cord. However, due to the extreme complexity and heterogeneity of brain structure and function, most existing treatments have been unsatisfactory. As mentioned above, during the pathological processes of AD, PD, and MS, activated platelets facilitate the development of these diseases, and recent studies have found that antiplatelet drugs have therapeutic effects on the aforementioned conditions.

#### 6.1.1 Aspirin

Aspirin (acetylsalicylic acid) is an excellent choice for targeting both platelets and neurons, as it irreversibly inhibits platelet COX enzymes, leading to the suppression of TXA2 synthesis, which results in platelet aggregation and vasoconstriction. In patients with AD, aspirin can activate peroxisome proliferator-activated receptor alpha (PPARα) by increasing lysosomal activity within brain cells, thereby reducing Aβ formation and regulating hippocampal synaptic plasticity, which enhances learning and memory (Chandra et al., 2018; Patel et al., 2018). Research by Thrash-Williams et al. (2016) has shown that salicylates significantly reduce methamphetamine-induced death of dopaminergic neurons by inhibiting mitochondrial dysfunction and clearing ROS, in addition to improving motor impairments under PD conditions. In a recent study, Fyfe (2020) also suggested a potential role for aspirin in reducing the risk of PD related to *LRRK2* expression. Other studies have found that aspirin can reduce CD4 T-lymphocyte infiltration into the CNS, decrease levels of pro-inflammatory factors such as TNF-α and IL-1β, alleviate symptoms of EAE, and positively impact treatment for MS (Vogelsang et al., 2021; Tsau et al., 2015). However, treatment with aspirin is not without risk, with an increased risk of cerebral hemorrhage and cerebral hypoperfusion in addition to duodenal toxicity. In the ASPREE trial, instead of reducing ischemic stroke, daily low-dose aspirin increased the risk of intracranial hemorrhage by 38% (Cloud et al., 2023).

Therefore, aspirin may serve as a potential therapeutic agent for NDDs, but it should be used with caution in older adults who are prone to head trauma (e.g., falls).

#### 6.1.2 Cilostazol

Cilostazol, an effective antiplatelet agent and phosphodiesterase III (PDEIII) inhibitor, can inhibit the activity of PDE, obstructing the degradation of cyclic adenosine monophosphate (cAMP) and preventing the dephosphorylation of vasodilator-stimulated phosphoprotein (VASP; Coenen et al., 2021). It possesses antiplatelet aggregation properties, protects endothelial cells (Xue et al., 2022), promotes angiogenesis (Su et al., 2019), and can directly dilate blood vessels and modulate inflammatory responses, thereby preventing early deterioration of neural function (Chen et al., 2021). Cilostazol suppresses Aβ-induced neurotoxicity via ROS-activated mitogen-activated protein kinase (MAPK)-p38 signaling and AMPK/CREB pathways, demonstrating neuroprotective effects against Aβ-induced neurodegeneration and inhibiting Aβ formation to improve cognitive decline in AD (Ono and Tsuji, 2019). It can upregulate the expression of nuclear receptor coactivator 1 induced by rotenone in PD rats, preserving the function and integrity of dopaminergic neurons while inhibiting the expression of pro-inflammatory molecules such as NF-κB, TNF-α, and IL-1β to reduce neuroinflammation (Hedya et al., 2018).

#### 6.1.3 Clopidogrel and ticagrelor

Clopidogrel and ticagrelor act on the P2Y12 receptor on platelets and are classic antiplatelet drugs. Clopidogrel can improve learning and memory in aluminum chloride-induced AD model rats by reducing hippocampal acetylcholinesterase activity, TNF-α,IL-1β concentrations, and APP mRNA gene expression, thereby alleviating neuroinflammation and exhibiting neuroprotective effects (Khalaf et al., 2017). Ticagrelor improves cerebral blood flow and neuroprotection through the phosphorylation of endothelial nitric oxide synthase (eNOS) and ERK1/2 (Yamauchi et al., 2017). A meta-analysis of randomized controlled trials suggests that ticagrelor may provide more favorable outcomes for all stroke, ischemic stroke, and transient ischemic attack prevention in patients with vascular risk factors. However, this benefit may be accompanied by costs of intracranial hemorrhage, dizziness, and insomnia. Tegretol may reduce the risk of dementia and Parkinson's disease, although available data are limited (Li et al., 2022). More interestingly, clopidogrel and ticagrelor, two P2Y12-specific antagonists, effectively alleviated the disease severity of EAE and inhibited Th17 differentiation both *in vivo* and *in vitro*.

But it also indicates that high doses of clopidogrel can cause more severe symptoms of EAE (Qin et al., 2017). Given that stroke is considered a significant vascular risk factor for developing PD, ticagrelor has been proposed to lower the risk of vascular complications in PD patients, including stroke and dementia (Li et al., 2022). In this regard, clopidogrel and ticagrelor may represent new therapeutic candidates for the treatment of NDDs, but should be used with caution regarding dosage and administration.

#### 6.1.4 Dipyridamole

Dipyridamole can increase the concentration of cAMP in platelets by inhibiting phosphodiesterase activity, inhibit platelet aggregation, and increase the concentration of cAMP in vascular smooth muscle cells, leading to vasodilation and increased peripheral arterial blood flow. In a recent report, Höllerhage et al. (2017) reported that dipyridamole can rescue neuronal death by reducing the release of lactate dehydrogenase (LDH) from overexpressed α-synuclein-containing neurons in the LUHMES, as well as reducing oxidative stress and promoting the expression of TH in PD. Dipyridamole reduced the expression of IL-1β, IL-6, IL-8, IL-12, MCP-1, IFN-γ, and TNF-α in lipopolysaccharide-induced microglia, and reduced the histological score of EAE, alleviating the symptoms of EAE (Sloka et al., 2013).

#### 6.1.5 Dabigatran etexilate

Dabigatran is a direct thrombin inhibitors that specifically prevents thrombin-stimulated platelet activation and aggregation in a dose-dependent manner. Dabigatran etexilate can improve the symptoms of AD both in the long and short term. It can inhibit the formation of thrombin and occlusive thrombus in AD, inhibit oxidation and inflammation, improve neuroinflammation and amyloid deposition in AD mice, and protect cognitive, cerebral perfusion, and blood-brain barrier functions (Cortes-Canteli et al., 2019; Iannucci et al., 2020). Furthermore, treatment with dabigatran restored dopamine levels by enhancing Nur-related factor 1 (Nurr1) expression and increasing the transcriptional activation of TH, vesicular monoamine transporter VMAT, and glial cell line-derived neurotrophic factor (GDNF), significantly reducing rotenone-induced neurodegeneration. In rotenone-induced PD rats, it also inhibited the accumulation of thrombin and α-synuclein in the substantia nigra, subsequently reducing neuroinflammatory markers such as NF-?B and TNF-α (Kandil et al., 2018). In addition, a study showed that dabigatran inhibited the thrombin effect of astrocytes by reducing protease-activated receptor-1 (PAR1) activation, downregulating the sphingosine kinase 1 (SphK1) signaling pathway, and weakening the sphingosine-1-phosphate (S1P) receptor pathway. In the EAE model, it can effectively restore neural function, prevent spinal cord inflammation, and further reduce spinal cord demyelination (Chen et al., 2020).

### 6.2 Platelets as a candidate drug for the treatment of NDDs

Platelet products can promote therapeutic responses in multiple medical fields, including the CNS. Platelet-rich plasma (PRP) is a platelet concentrate obtained from autologous whole blood after centrifugation, containing a large amount of growth factors and proteins. In the AD mouse model, intranasal administration of PRP has neuroprotective effects, which may be mediated by activating the anti-apoptotic PIEK/Akt signaling pathway (Anitua et al., 2013). Farid et al. (2022) proved that PRP may have CNS protection and neurotrophic effects, thus achieving successful therapeutic effects in the treatment of NDDs. The characteristics of PRP can vary greatly depending on the age of the donor, with older donors having a stronger inflammatory profile. PRP can reduce the apoptosis of neural progenitor cells, stabilize neuronal synapses, and alleviate microglia inflammation. Additionally, young PRP has low levels of inflammatory molecules (Delgado et al., 2021). Intrathecal injection of PRP in EAE mice can significantly improve neurological function, manifested by a reduction in the proliferation area of astrocytes and microglia, a reduction in inflammatory cell infiltration, and downregulation of the expression of pro-inflammatory cytokines (Borhani-Haghighi and Mohamadi, 2019).

A recent study published in Nature showed that injecting young mouse plasma into old mice can promote the molecular level increase of synaptic plasticity, which changes the ability of neural cells to connect with each other, reduce brain inflammation, and ultimately enhance cognitive ability (Schroer et al., 2023). After injection of “longevity factor” Klotho, platelet activation leads to the release of Platelet factor 4 (PF4) and other factors, enhancing cognitive ability in young and old animals, and also making the brain more resistant to age-related degeneration (Park et al., 2023). Exercise can improve brain health through platelets (Leiter et al., 2023). These three studies indicate that brain health and cognitive enhancement are closely related to platelets, and are also closely related to platelet factor 4 (a specific protein synthesized by platelet alpha-granules).

Therefore, it is possible that platelets in a non-diseased and young state can promote tissue regeneration and improve brain function by releasing various growth factors.

## 7 Challenges and future outlook

With the progress of population aging, the prevalence of NDDs is increasing year by year. Many of the means and methods for diagnosing NDDs rely on invasive research, which causes certain trauma to the human body. In this regard, it is necessary to discover peripheral markers for NDDs. Although significant progress has been made in studying the structural, functional, and secretory changes of platelets in NDDs, existing research has limited research on peripheral specific biomarkers for NDDs, so these findings still provide possibilities for future research in this field. The neuroprotective potential of antiplatelet drugs has been intensively studied for many years. Although current research has preliminarily revealed the potential role of platelets in NDDs, there is still controversy regarding the lack of clinical evidence for NDDs treatment procedures, as most data on the efficacy of antiplatelet drugs in NDDs come from laboratory studies. Antiplatelet drugs may be additional drugs or combination therapy, and can be used as adjuvants to established classic drugs to improve the efficacy of existing drugs and better to treat NDDs. Due to the ability of young and healthy platelets to secrete various growth factors and play an important role in neuroprotection, the most obvious challenge in the treatment of NDDs with single or dual anti-platelet regimens is the degree of platelet inhibition. Determining the optimal dose and timing, and restoring the normal state of platelets, is an effective treatment prospect for the treatment of NDDs.

## 8 Conclusions

Platelets are a special type of blood cell in the blood circulation. Due to their small size, high number, and rapid turnover, as storage and secretory cells, they play a complex and important role in NDDs. Activated platelets are significantly similar to neurons, participating in blood coagulation and thrombosis, enhancing inflammatory response and oxidative stress to promote disease progression, and serving as potential peripheral diagnostic biomarkers and therapeutic targets for AD, PD, and MS. Hence, with the emergence of new technologies, platelet-based detection and therapy may represent a possible future direction for the diagnosis and treatment of NDDs.
